# Non-Host Volatile Blend Optimization for Forest Protection against the European Spruce Bark Beetle, *Ips typographus*

**DOI:** 10.1371/journal.pone.0085381

**Published:** 2014-01-14

**Authors:** C. Rikard Unelius, Christian Schiebe, Björn Bohman, Martin N. Andersson, Fredrik Schlyter

**Affiliations:** 1 Department of Chemistry and Biomedical Sciences**,** Linnaeus University, Kalmar, Sweden; 2 Department of Plant Protection Biology, Swedish University of Agricultural Sciences, Alnarp, Sweden; Rutgers University, United States of America

## Abstract

Conifer feeding bark beetles (Coleoptera, Curculionidae, Scolytinae) pose a serious economic threat to forest production. Volatiles released by non-host angiosperm plants (so called non-host volatiles, NHV) have been shown to reduce the risk of attack by many bark beetle species, including the European spruce bark beetle, *Ips typographus*. However, the most active blend for *I. typographus*, containing three green leaf volatiles (GLVs) in addition to the key compounds *trans*-conophthorin (tC) and verbenone, has been considered too expensive for use in large-scale management. To lower the cost and improve the applicability of NHV, we aim to simplify the blend without compromising its anti-attractant potency. Since the key compound tC is expensive in pure form, we also tested a crude version: technical grade *trans*-conophthorin (T-tC). In another attempt to find a more cost effective substitute for tC, we evaluated a more readily synthesized analog: dehydro-conophthorin (DHC). Our results showed that 1-hexanol alone could replace the three-component GLV blend containing 1-hexanol, (3*Z*)-hexen-1-ol, and (2*E*)-hexen-1-ol. Furthermore, the release rate of tC could be reduced from 5 mg/day to 0.5 mg/day in a blend with 1-hexanol and (–)-verbenone without compromising the anti-attractant activity. We further show that T-tC was comparable with tC, whereas DHC was a less effective anti-attractant. DHC also elicited weaker physiological responses in the tC-responding olfactory receptor neuron class, providing a likely mechanistic explanation for its weaker anti-attractive effect. Our results suggest a blend consisting of (–)-verbenone, 1-hexanol and technical *trans*-conophthorin as a cost-efficient anti-attractant for forest protection against *I. typographus*.

## Introduction

**Figure 1:**
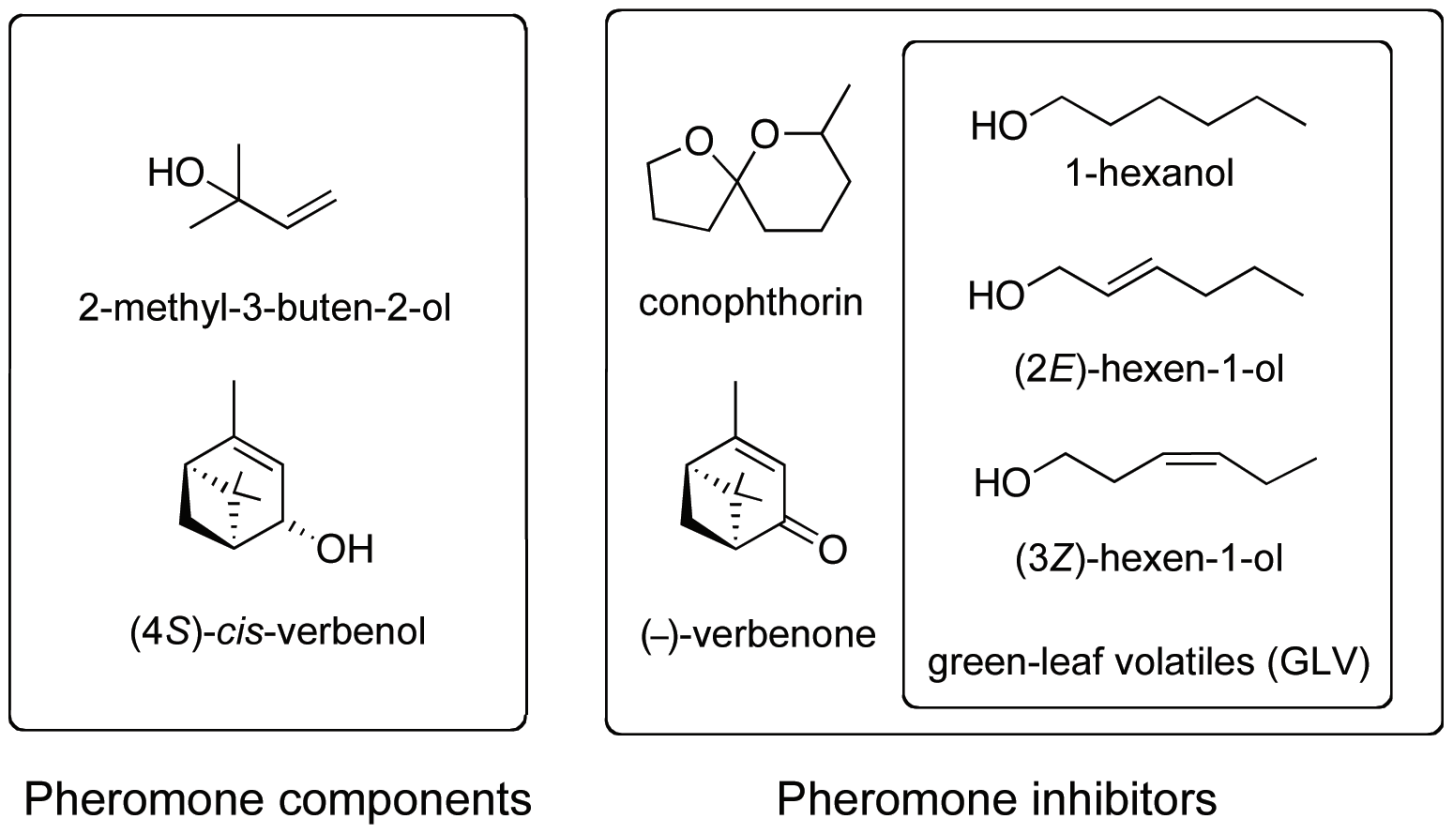
Structural formula of pheromone compounds and pheromone inhibitors included in the non-host volatile blends.

Bark beetles (Coleoptera, Curculionidae, Scolytinae) are a major concern for the forest industry, causing large economic losses during outbreak periods. Typically, the beetles utilize aggregation pheromones to coordinate mass-attacks on coniferous trees, normally resulting in the death of the tree and sometimes large-scale forest destruction [1]. Volatiles released by non-host angiosperm plants, so called non-host volatiles (NHV), have been shown to inhibit pheromone attraction of several coniferous bark beetle species and are proposed to guide beetles in search for host trees [2]–[7]. The beetle response to NHV offers a new and promising approach for the protection of coniferous forest stands from attack. In addition to NHV, oxygenated monoterpenes from attacked trees seem to be an important cue for discrimination between suitable or overpopulated hosts [8]–[10]. For example (–)*-*verbenone, the oxygenation product of the host terpene (–)-α-pinene and the pheromone component (4*S*)-*cis*-verbenol [11], is found in gallery walls of Norway spruce, *Picea abies* (L.) H. Karst, after infestation by the European spruce bark beetle, *Ips typographus* (L.) (Fig. 1) [12], [13], the most serious bark beetle pest in Europe. This compound inhibits attraction to the aggregation pheromone [14], [15], which is a mixture of (4*S*)-*cis*-verbenol and 2-methyl-3-buten-2-ol (Fig. 1) [13]. Verbenone alone is applied in the control of American *Dendroctonus* species [16]–[18] and has also been tested in experiments to protect spruce trees and logs from attack by *I. typographus*. Used alone however, it is only weakly inhibitory for *I. typographus* [19]. Fortunately, its inhibitory effect on *I. typographus* is strongly synergized by NHV, such as the green leaf volatiles (GLVs) (3*Z*)-hexen-1-ol, (2*E*)-hexen-1-ol, 1-hexanol, as well as *trans*-conophthorin (tC) [20]. The latter compound, [(E)-7-methyl-1,6-dioxaspiro[4.5]decane] is found in bark of deciduous trees, e.g. birch, (Fig. 1) [20].

At present there is no effective method to control the large-scale and recurring outbreaks of *I. typographus* in spruce forests. However, we recently showed that groups of spruce trees, especially at exposed forest edges, could be protected by a commercial product called IT-REP (Fytofarm Ltd., Bratislava, Slovakia) that consists of a wick dispenser-pouch containing verbenone, GLVs, 1-octen-3-ol, 3-octanol and tC [21]. Overall, a meta-analysis based on all papers published 2000–2012 for two of the world’s most noxious bark beetle pests, *I. typographus* and the mountain pine beetle (*Dendroctonus ponderosae*) showed a strong effect of NHV and verbenone against mass attack [22].

In order to successfully apply NHV in large-scale management of *I. typographus* or other bark beetle species, there is a need for more cost-effective preparation of the anti-attractant compounds and mixtures. In particular, the most active NHV blend for *I. typographus* contains many compounds [4], some of which are expensive to synthesize. In addition, the expensive key compound tC has hitherto been synthesized only on a small scale and has not been commercially available.

**Figure 2:**
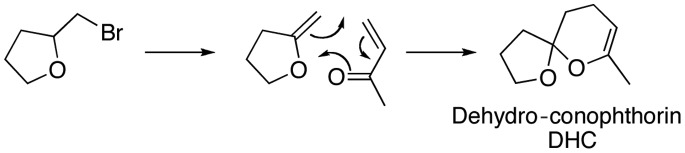
Synthesis of racemic dehydro-conophthorin.

By a combination of chemical synthesis, electrophysiology, and field trapping studies, we investigated if the use of NHV against *I. typographus* could be made more economically feasible. First, we tested whether 1-hexanol alone could be a substitute for the expensive three-component GLV blend, containing (3*Z*)-hexen-1-ol and (2*E*)-hexen-1-ol in addition to 1-hexanol [4]. We also determined the minimum release rate of tC required to obtain a synergistic reduction in pheromone trap catch when tC was included in a multicomponent NHV mixture. We then tested a less expensive preparation of the tC, i.e. technical grade tC (T-tC), which is now commercially available (Table 1). The inhibitory effect of T-tC on *I. typographus* pheromone trap catch was compared to the effect of pure tC. Furthermore, by using inexpensive and commercially available precursors, we synthesized a more easily prepared analog to tC: dehydro-conophthorin (DHC, Fig. 2). DHC was also tested for anti-attractive effects in field trapping experiments and compared with the performance of tC. To mechanistically elucidate the behavioral differences found, the physiological sensitivity of the tC-responsive olfactory receptor neuron (ORN) class [23] to tC, T-tC and DHC was studied using single-sensillum recordings (SSR). Based on our results, we propose multiple ways of simplifying the anti-attractant blend and reducing its cost without compromising efficiency and we provide a physiological mechanism in the peripheral olfactory sense that is likely to explain our observations.

**Table 1:** Compounds and release rates used in the three trials.

	Manufacturer and purity	Release rate(mg/day)	Compound amount and dispenser	Ref.
***Pheromones***				
2-methyl-3-buten-2-ol	Acros Organic, New Jersey, USA, 97%	57	2 mL in #733 PE-vial with 2 mm hole in the lid	[13]
(4*S*)-(−)-*cis*-verbenol	Fytofarm, Bratislava, Slovakia, 98%	0.1	50 µg in #730 PE-vial with 5 mm of a150 µL- capillary in lid	[13]
IT ECOLURE Tubus	Fytofarm, Bratislava, Slovakia	95	Closed PE tube	[25]
*Ips typographus* lure	Chemtica Int. S.A., Costa Rica	61	PE pouches	a
***Inhibitors***				
(−)-verbenone	Bedoukian, Danbor, USA, 90%	0.5	150 µL in open #730 PE-vial	[4]
1-hexanol	Sigma-Aldrich, Germany, 98%	6	2×300 µL in open #730 PE-vial	[42]
1-hexanol	Sigma-Aldrich, 98%	60	1.2 mL in open #731 PE-vial	[42]
GLV: 1-hexanol(3*Z*)-hexen-1-ol,(2*E*)-hexen-1-ol	Sigma-Aldrich, 98%Acros Organic, 98%Fluka, Germany, 98%	6	200 µl of 1∶1∶1 mix in open #730 PE-vial	[42]
*trans*-conophthorin	Syntagon, Sweden, 97%	0.05	5 µL-Microcaps® glass capillary	[42]
*trans*-conophthorin	Syntagon, Sweden, 97%	0.5	2×44.7 µL-Microcaps®	[42]
*trans*-conophthorin	Syntagon, Sweden, 97%	5	150 µL in open #730 PE-vial	[4]
dehydro-conophthorin	Synthesized, see exp.	0.5	2×44.7 µL-Microcaps®	a
Tech. *trans*-conophthorin	Syntastic AB^b^, Sweden, >70% (GC)	0.5^c^	2×44.7 µL-Microcaps®	a
Tech. *trans*-conophthorin	Syntastic AB, Sweden, >70% (GC)	5^c^	150 µL in open #730 PE-vial	a

#730 PE-vial: Polyethylene vial with 6 mm ID, 29 mm height (Kartell, Italy). #731 PE-vial: Polyethylene vial with 13 mm ID, 24 mm height (Kartell, Italy). #733 PE-vial: Polyethylene vial with 20 mm ID, 29 mm height (Kartell, Italy). Microcaps® glass capillaries were sealed at one end with dental wax.

^a^ Measured in the present study by weight reduction over time in fume hood.

^b^ http://www.syntastic.se/.

^c^ Release rates of T-tC were not corrected for presence of impurities.

## Materials and Methods

### Synthesis of DHC

^1^H and ^13^C NMR spectra were recorded at 298 K at 500 and 125 MHz on a Varian Unity 500 instrument. Chemical shifts are reported in ppm. NMR experiments were run in deuterated chloroform (CDCl_3_) and are referenced to the resonance from residual CHCl_3_ at 7.26 ppm for ^1^H and to the central peak in the signal from CDCl_3_ at 77.0 ppm for ^13^C. The appearance and multiplicities of ^1^H resonances are expressed by the abbreviations: s (singlet), m (multiplet) and br (broad) and described by chemical shift (multiplicity, integration).

EI-GCMS (70 eV) were recorded on an Agilent 5973 mass detector connected to an Agilent 6890 GC equipped with a BPX-70 column (30 m×0.25 mm×0.25 µm film thickness, SGE Australia), using helium as carrier gas. Mass/charge ratios (*m/z*) are reported and relative abundances of the ions as percentages of the base peak intensity.

#### 2-Methylenetetrahydrofuran

Tetrahydrofurfuryl bromide (4.36 g, 3.00 mL, 26.6 mmol, tech. grade 90% from Alfa Aesar) was added to powdered KOH (6.0 g, 107 mmol) at 0°C. The viscous mixture was stirred occasionally during 30 min, after which the mixture was allowed to equilibrate to room temperature (RT). The product was isolated by simple distillation and 1.56 g (78% yield, calculated on 90% purity of starting material) colorless oil was collected over K_2_CO_3_. The product (47% yield, 61% purity by GC-MS) was used as such in the next step. EI-GCMS: 39(6), 41(11), 42(7), 43(19), 44(2), 53(2), 55(6), 71(100), 72(5), 93(2), 95(2), 121(2), 123(2).

NMR data were in accord with the literature [24].

#### Dehydro-conophthorin (DHC)

2-Methylenetetrahydrofuran (210 mg, 2.50 mmol), methyl vinyl ketone (175 mg, 2.50 mmol, 99% purity, Aldrich), and hydroquinone (ca 10 mg, 99% purity, Fluka) were added to a vial, which was closed and stored at RT for 6 days. The product was dissolved in ether, washed with water (10 mL), brine (10 mL), dried over MgSO_4,_ and the ether was removed *in vacuo* yielding a yellow, fragrant oil (0.27 g, 71% yield). GC-MS analysis proved the purity to be 89% (total yield 63%). EIMS 41(6), 42(10), 43(12), 55(8), 56(14), 71(5), 84(100), 85(6), 97(5), 154(25). ^1^H NMR δ 4.55(m, 1H), 4.07(m, 1H), 3.96(m, 1H), 2.08–2.26 (m, 3H), 1.90–2.04 (m, 2H), 1.72–1.85 (m, 3H), 1.71 (br s, 3H). ^13^C NMR δ 148.78, 106.60, 95.46, 68.29, 36.85, 29.96, 24.27, 20.47, 19.01.

### Trapping Experiments

#### Traps and baits

No permissions were required for the use of traps together with bark beetle specific pheromones. Pipe traps (12.5 cm cylinder diameter, 1.4 m high) [3] and Lindgren multiple-funnel traps (10-units Phero Tech Inc., Canada) were used in 2006 and 2007 experiments (Trial 1 and 2), while only pipe traps were used in the 2008 experiment (Trial 3). Dispensers with pheromone and anti-attractants were either hung inside pipe traps or placed under an inverted 250 mL light grey, plastic cup (to protect chemicals from sun and weather exposure) and attached to Lindgren traps. All chemicals used and their release rates are provided in Table 1.

In 2006 (Trial 1) we released the two *I. typographus* aggregation pheromone components, 2-methyl-3-buten-2-ol and (4*S*)-*cis*-verbenol, from separate polyethylene vials (Table 1). In 2007 (Trial 2) we applied commercial dispenser pouches of *Ips typographus* lure (Chemtica International S.A., Costa Rica). In 2008 (Trial 3) we applied commercial IT Ecolure Tubus (Fytofarm, Slovakia) as attractant [25].

#### Field trials

In all experiments the dispensers were rotated among traps counter-clockwise between each replicate, so that each treatment was positioned at least once at each trap position (“temporal” Latin square design). Trap catches were collected and treatments rotated once ≥20 beetles were caught in at least one trap in each row, which defined one replicate.

**Figure 3:**
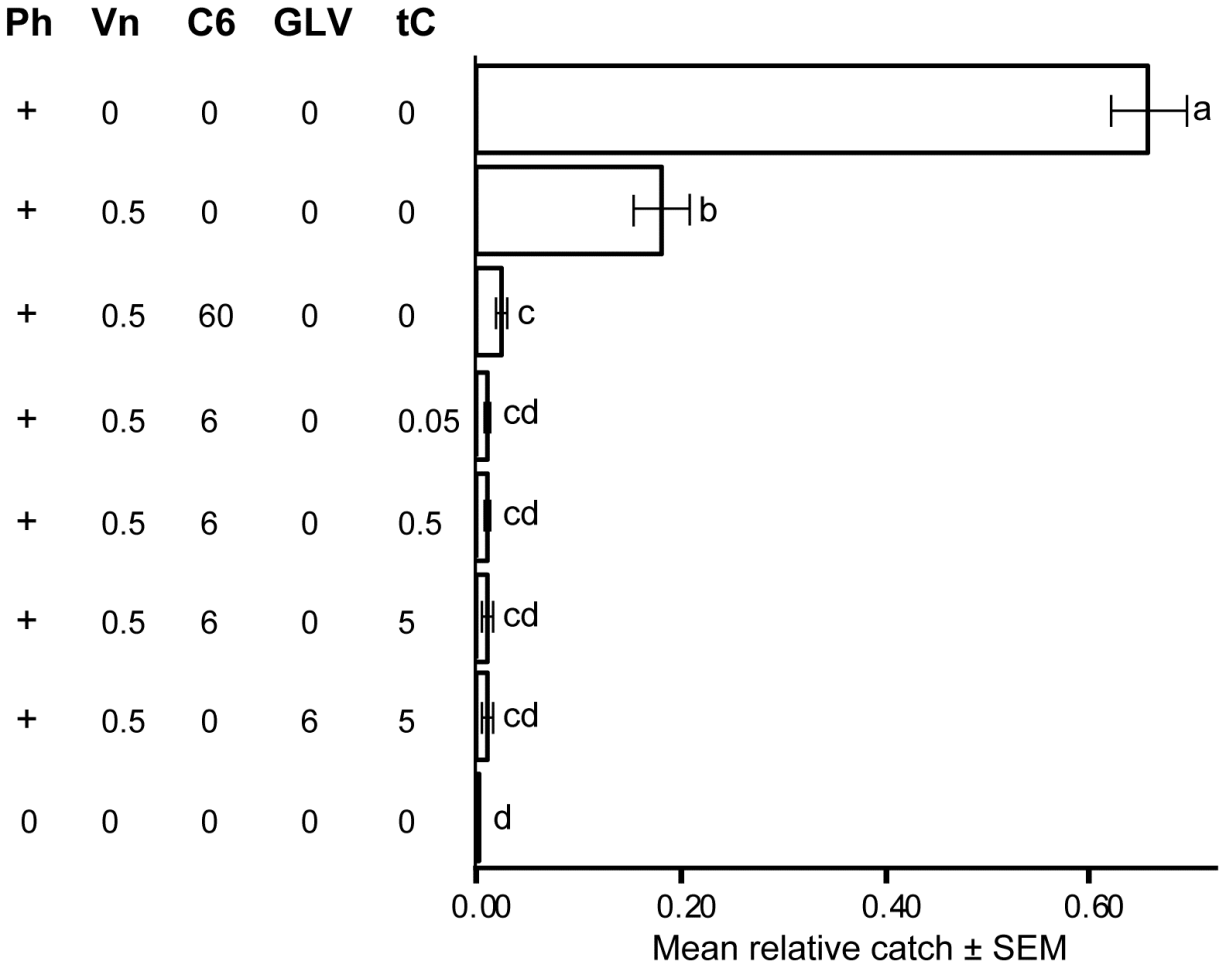
Reducing the release of *trans-*conophthorin and comparing a GLV-blend with 1-hexanol alone, Trial 1. Results from field experiment in Asa, South Sweden, May − June 2006. Bars show mean relative catch per replicate ±1 standard error based on *n = *21 replicates (trap rotations). Numbers below stimuli acronyms are release rates (mg/day; Table 1). Acronyms: Ph (+) = *Ips typographus* pheromone blend (ca 57 mg/day); Vn = (–)-verbenone; GLV = (3*Z*)-hexen-1-ol, (2*E*)-hexen-1-ol, 1-hexanol (6 mg/day for a 1∶1∶1 mixture), C6 = 1-hexanol; tC = *trans*-conophthorin. Bars with same letters are not significantly different (ANOVA on arcsin√(relative catch), followed by Dunnet’s T3 multiple range post-hoc test, p<0.05).

Trial 1 (Fig. 3): The field trial in 2006 was performed during May and June in a clear cut area (ca. 1.8 ha) near Asa forest research station in southern Sweden (N 57°11′ E 14°49′), owned by the Swedish state’s forest company Sveaskog, from which permission for the experiment was received. The population of *I. typographus* was low, but rising due to a large number of wind-thrown trees in the region following a storm the previous year [26]. This experiment was designed to investigate if the dose of tC could be reduced without compromising efficiency of the anti-attractant blend, and also to test whether 1-hexanol alone could replace a three-component blend of 1-hexanol, (3*Z*)-hexen-1-ol, and (2*E*)-hexen-1-ol, previously deployed by Zhang & Schlyter [4]. A pheromone only trap and a blank (unbaited) trap were included as controls. Six treatments with anti-attractants were tested: the first treatment included the pheromone and the anti-attractant (–)-verbenone (0.5 mg verbenone/day, i.e. the release rate for verbenone in all trials) (Table 1 and Fig. 3) [4], [27]. The second treatment had pheromone plus 1-hexanol (60 mg/day) and (–)-verbenone as anti-attractants. This treatment was included to test if tC could be replaced by (–)-verbenone and a ten times higher release rate of 1-hexanol than previously used [3]. In three of the treatments, pheromone plus (–)-verbenone and 1-hexanol were tested in combination with three release rates of tC (0.05, 0.5, and 5 mg/day, respectively). Finally, a sixth treatment was a positive control that was the previously tested three-component GLV blend [3], [4] at its original release rates (6 mg/day for the three compounds combined) combined with tC (5 mg/day) and (–)-verbenone. Two sets of the eight differently baited traps were placed in two rows. One set consisted of pipe traps and the other of Lindgren multiple-funnel traps. The minimum distance was 10 m between traps and 25 m between traps and the forest border. Baits in pipe traps were rotated 13 times (each rotation defining a replicate), whereas baits in Lindgren traps were rotated 8 times before flight activity ceased.

**Figure 4:**
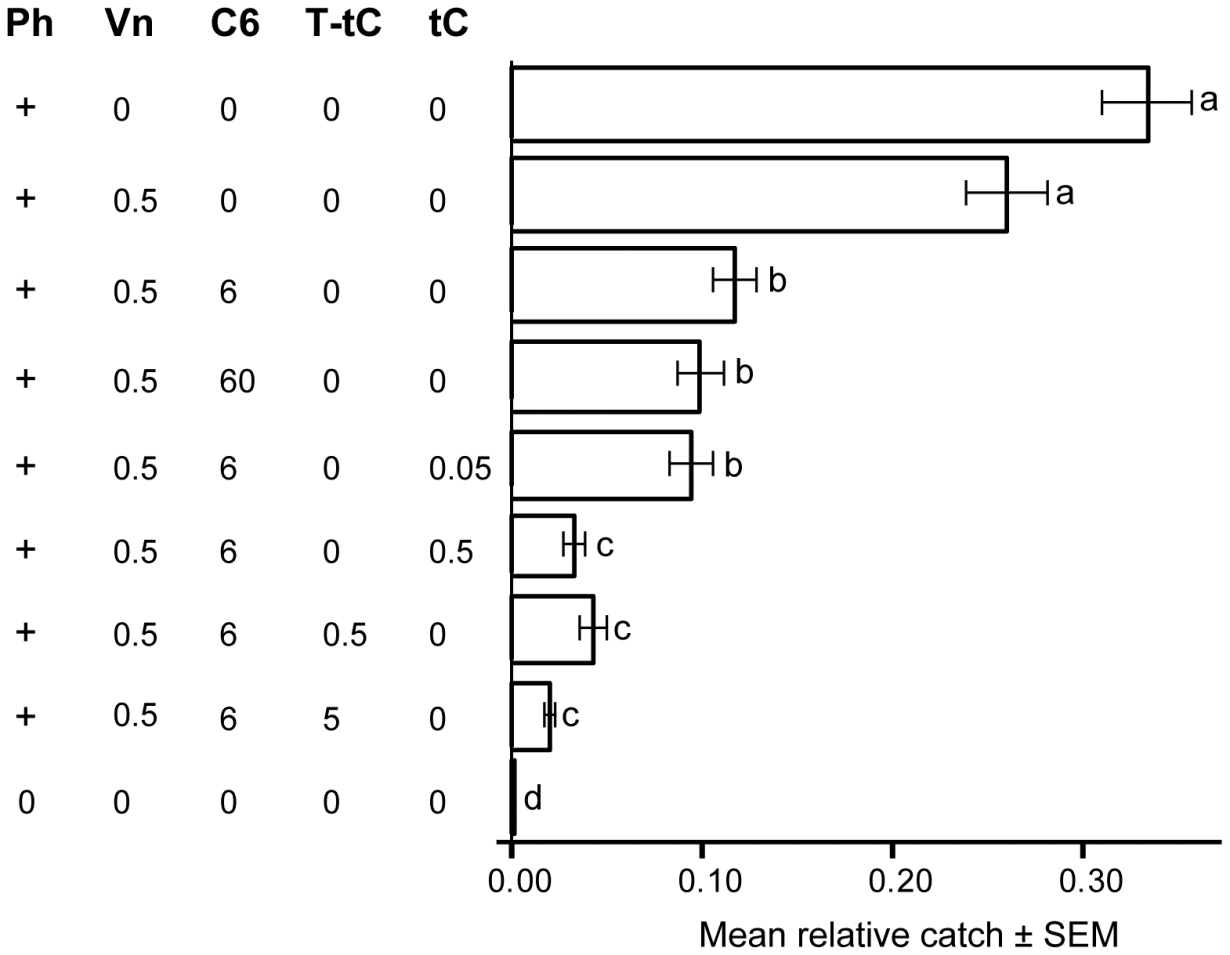
Comparison of trap catch reduction by *trans*-conophthorin versus technical grade *trans*-conophthorin, Trial 2. Results from field trapping test in Germundslycke, May – June 2007. Bars show mean relative catch per replicate ±1 standard error based on *n = *81 (*n = *80 for Vn; *n = *79 for C6 at 60 mg/day) replicates. Numbers below stimuli acronyms are release rates (mg/day; Table 1). Acronyms: Ph = pheromone (commercial lure from Chemtica); Vn = (–)-verbenone; C6 = 1-hexanol; tC = *trans*-conophthorin; T-tC = technical grade *trans*-conophthorin. Bars with same letters are not significantly different (ANOVA on arcsin√(relative catch) followed by Dunnet’s T3, p<0.05).

Trial 2 (Fig. 4): The experiment in 2007 was performed from 15 May to 19 July in Germundslycke in South East Sweden (N 56°31′E 16°03′) in a privately owned area (see acknowledgements) cleared from trees after a heavy bark beetle outbreak the previous year. Permission for the experiment was granted by the owner. In this test, we primarily aimed to investigate the inhibitory effect on trap catch of pure tC compared to that of T-tC. Two lines with pipe traps were placed with >10 m between traps and one line with Lindgren multiple-funnel traps was placed perpendicular to the two other lines. Each line consisted of nine traps including seven treatments with anti-attractants, a blank control trap, and a trap baited with only pheromone as a second control (Fig. 4, Table 1). The first treatment included pheromone and (–)-verbenone. The second had pheromone plus (–)-verbenone and 1-hexanol (6 mg/day) as anti-attractants. The third treatment had the same compounds as the second treatment, but a ten times higher release (60 mg/day) of 1-hexanol. The forth and fifth treatments included 0.05 mg/day or 0.5 mg/day of pure tC, respectively, in addition to 1-hexanol and (–)-verbenone. The sixth treatment included T-tC (0.5 mg/day) instead of tC, but was otherwise identical to treatment five. Finally, the seventh treatment had a ten times higher release rate (5 mg/day) of T-tC to test whether a contingently lower efficiency of T-tC could be compensated for by a higher release rate. The lowest tested release rate of tC (0.05 mg/day; fourth treatment) and the ten times higher rate of 1-hexanol without tC (60 mg/day; third treatment) were included here to verify the results from Trial 1 at higher beetle population levels. All baits in a line were tested three times at each position in that line, resulting in a total of 27 replicates for each line and a grand total of 81 replicates for the three lines together.

**Figure 5:**
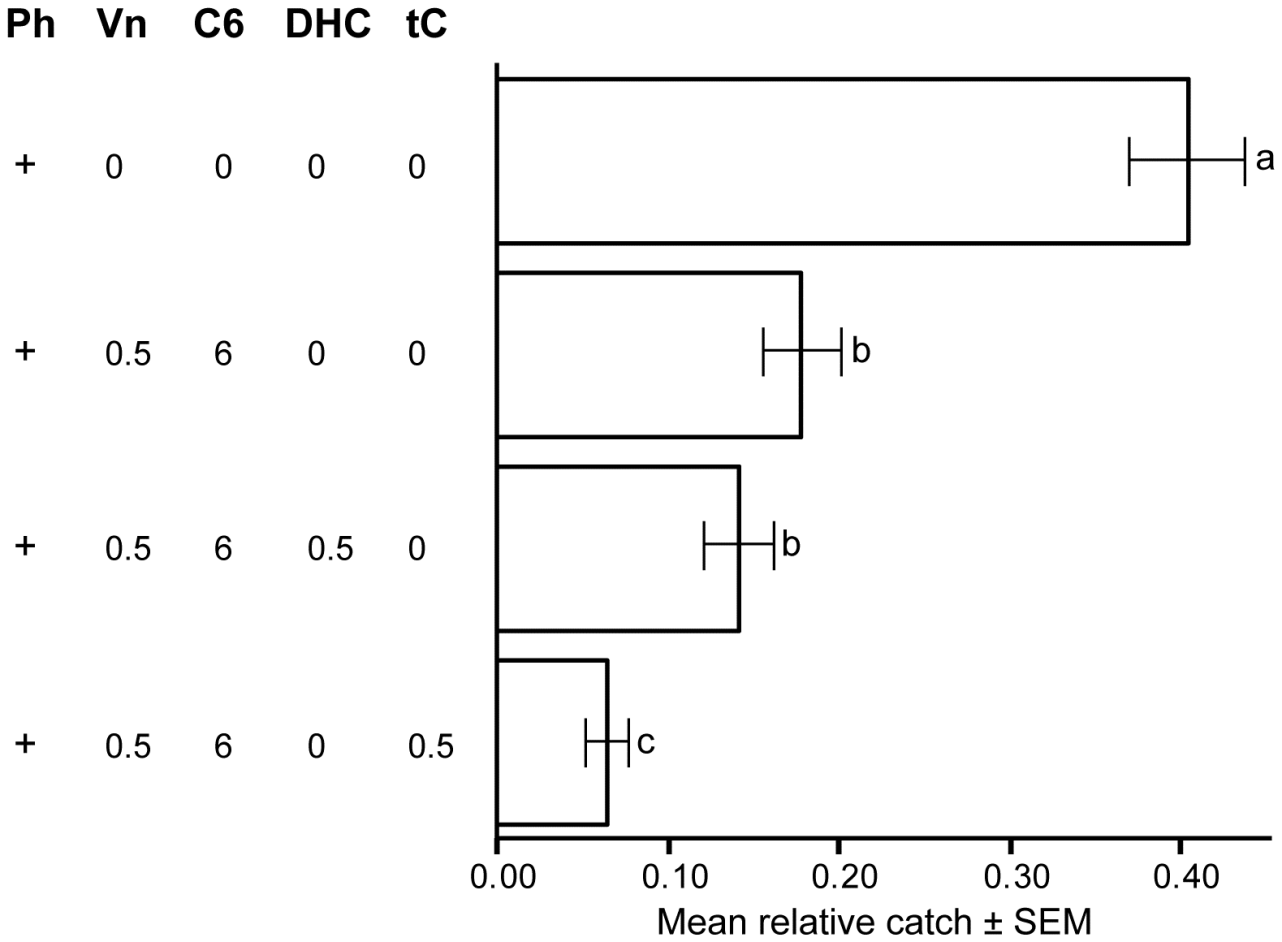
Test of dehydro-conophthorin, Trial 3. Results from field trapping test in Parismåla, May 2008. Bars show mean relative catch per replicate ±1 standard error based on *n = *15 replicates. Numbers below stimuli acronyms are release rates (mg/day). Acronyms: Ph = pheromone (IT Ecolure Tubus, Fytofarm, Slovakia); Vn = (–)-verbenone; C6 = 1-hexanol; tC = *trans*-conophthorin; DHC = dehydro-conophthorin. Bars with same letters are not significantly different (ANOVA on arcsin√(relative catch) followed by Tukey’s HSD multiple range post-hoc test, p<0.05).

Trial 3 (Fig. 5): The experiments in 2008 were performed during May in Parismåla, Southeast Sweden (N 56°35′ E 15°28′) in a privately owned forest (see acknowledgements). Permission for the experiment was granted by the owner. The regional bark beetle population density was high following two years of numerous mass attacks. To study whether DHC had inhibitory effects on pheromone trap catch, we used one row with four pheromone-baited pipe traps separated by at least 10 m. Traps were baited in the following way: a pheromone alone control; pheromone plus 1-hexanol and (–)-verbenone; pheromone plus 1-hexanol, (–)-verbenone, and DHC; and finally pheromone plus 1-hexanol, (–)-verbenone, and tC. DHC and tC were released at 0.5 mg/day, 1-hexanol at 6 mg/day. A total of 15 rotations (replicates) were completed in this Trial.

#### Analysis of trap catch data

Relative catch was the basic variable for statistical processing and was calculated for individual traps by dividing the number of beetles caught by the total catch (in all traps) for that replicate. No differences were found between the two different trap types (pipe traps and multiple-funnel traps) used, neither in Trial 1 (ANOVA on arcsin√(relative catch) transformed catch values: F = 0.17, p = 0.68) nor in Trial 2 (F = 0.51, p = 0.6), and thus treatments from different trap types were pooled.

In Trials 1 and 2 the variation in catches between different treatments was high including several outliers and there was consistently no or very low catch in blank traps. In order to compensate for this variation, several transformations of relative trap catch were performed and the most equal variances were achieved by arcsin√(relative catch) transformation. However, since this transformation still did not result in homogenous variances as indicated by Levene and Welsh tests, we used Dunnet’s T3 for p<0.05 as multiple range post-hoc test allowing comparisons with unequal variances following the ANOVA. In Trial 3 transformation by arcsin√(relative catch) resulted in homogeneous variances and Tukey HSD was used as Post-hoc test. All statistical analyses were performed using SPSS 11.0 for Windows.

### Single-sensillum Recordings

Single-sensillum recordings with tungsten microelectrodes were performed under a Nikon FN-S2N light microscope (500x magnification) using standard equipment (Syntech, Kirchzarten, Germany) and our experimental protocol [23]. The reference electrode was inserted through a pre-made hole in the pronotum and the recording electrode into the base of a sensillum. The antenna was exposed to a continuous charcoal-filtered and humidified airflow (1.2 L/min; controlled by stimulus controller CS-01, Syntech). The air was directed to the insect via a glass tube (7 mm i.d.) that terminated ca. 15 mm from the antenna.

Chemicals were diluted in hexane (Fluka, >99% purity) and 10 µl were applied on filter papers (ca. 1.5×0.5 cm) that were placed inside Pasteur pipettes. The solvent was allowed to evaporate before pipettes were capped with plastic pipette tips. Compound doses between 100 pg and 10 µg on the filter paper were tested in decadic steps, starting at the lowest dose. Stimulus pipettes were used for a maximum of two stimulations, as repeated stimulation rapidly reduces the airborne odor quantity [28]. At stimulation, a 0.5 s air-pulse at 0.2 L/min was passed through the stimulus pipettes and directed into the continuous airflow and, hence, to the antenna. The continuous airflow was simultaneously reduced by 0.2 L/min to ensure a constant total airflow over the antenna.

Neurons for *trans*-conophthorin were identified by comparing the response spectra of contacted ORNs to the previously determined response spectra of the tC ORN class [23]. As in our previous study, the tC ORN class was defined by a strong primary response to tC, and weaker responses to *exo-*brevicomin and chalcogran (and no other responses to the compounds tested by Andersson et al. [23]). Once electrical contact with a tC neuron was established, the neuron was tested for responses to pure (±)-tC, T-tC, and DHC. Both males and females (five sensilla from each) were used in recordings. Net olfactory responses were calculated by counting the number of spikes (in AutoSpike 3.0, Syntech) during the initial 0.5 s of the response and subtracting the number of spikes during the 0.5 s prior to the response. If the hexane control elicited response in a neuron, it was subtracted from the odor responses of that neuron.

The SSR dose-response data were analyzed using *Hedges’ g* standardized unbiased effect sizes and 95% confidence intervals (CI) [29]. At each dose, the response to pure tC was used as control group in pair-wise comparisons with T-tC and DHC. Thus, this analysis provided a measure of the physiological effect of T-tC and DHC in relation to the effect of the key stimulus (pure tC) of this neuron class. A response was regarded as significantly different from the response to pure tC when the CI did not include zero.

## Results

### Trial 1: Reducing tC Dose and the Number of GLV Compounds

A total of 2 625 *I. typographus* were caught in 21 replicates in Trial 1, which was designed to study whether the release rate of tC could be lowered and if 1-hexanol alone could replace a three-component GLV mixture without compromising the inhibitory effect of the anti-attractant blend. Catches among the two control traps and the six different inhibitory treatments were significantly different by ANOVA (on arcsin√(relative catch), F = 152.9; df = 7; p<0.001) followed by Dunnet’s T3. The pheromone control traps caught significantly (p<0.001) more beetles than all the other treatments (Fig. 3). Pheromone-baited traps with (–)-verbenone alone added caught significantly fewer beetles than the pheromone control (p<0.001), but significantly (p<0.001) more beetles than all treatments with NHV added to the combination of pheromone and (–)-verbenone. Combining 1-hexanol at 60 mg/day with (–)-verbenone significantly (p<0.001) reduced trap catches in comparison to the treatment with (–)-verbenone as the only inhibitor, but trap catches still remained significantly (p<0.01) higher than in blank traps. No significant differences were found between the individual treatments involving NHV. However, trap catch was reduced to a level not significantly different from that in blank traps only when tC was included. This indicated that tC needs to be included in the blend to achieve the strongest inhibition. We found no significant difference between the three release rates of tC. Thus, in this trial the treatment with 0.05 mg/day tC was as inhibitory as treatments with 10 or 100-fold higher tC release rates.

Finally, the treatment with 1-hexanol as the only GLV was not significantly different from the treatment with the three GLVs (1-hexanol, (3*Z*)-hexen-1-ol, and (2*E*)-hexen-1-ol) at corresponding release rates when included in the blend together with (–)-verbenone and tC.

The pheromone control traps had the highest proportion of males to females in the catch (45% males). Traps inhibited only with (–)-verbenone had 42% males, while trap baits including any additional NHV had 36% males in the catch. The difference between pheromone control, pheromone plus (–)-verbenone, or treatments with any NHV added, was significant (χ^2^ = 12.81; df = 2; p<0.05, based on counts of sexed beetles).

### Trial 2: Comparison between tC and T-tC

Trial 2 was conducted to compare the inhibitory activity on *I. typographus* trap catch of technical grade tC (T-tC) with that of pure tC, but some of the treatments from Trial 1 were also included for confirmation of the results from the previous year. A total of 34 208 beetles were caught in 81 replicates. Catches differed significantly between traps including the two controls and the seven inhibitory treatments as shown by ANOVA (F = 112.8; df = 8; p<0.001) followed by Dunnet’s T3. Traps baited with only pheromone had the highest catch (Fig. 4). In contrast to Trial 1, traps with (–)-verbenone as the only inhibitor did not reduce catches significantly compared to the pheromone control. However, adding 1-hexanol (at 6 or 60 mg/day; the two treatments were not significantly different from each other) as a second inhibitor in combination with (–)-verbenone significantly reduced trap catches (p<0.001), confirming this synergy from Trial 1.

Adding tC or T-tC at 0.5 or 5 mg/day in combination with 1-hexanol and (–)-verbenone significantly reduced trap catches further (p<0.001). This observation confirmed the results from Trial 1 regarding the importance of tC as a second synergist in reducing the attraction to the pheromone.

There was no significant difference in catch between traps with T-tC or tC included in the inhibitory blend, except for in the traps with the lowest release rate (0.05 mg/day) of tC. Thus, the addition of this lowest release rate of tC resulted in trap catches that were not significantly different from the treatments including only 1-hexanol and (–)-verbenone as inhibitors. Importantly, the lack of difference between tC and T-tC indicates that trap catch of *I. typographus* was neither influenced significantly by impurities in the technical grade treatment (T-tC), nor by the effectively lower release of tC from the technical grade preparation due to the ca. 28% lower tC content.

### Synthesis of DHC

The starting material 2-methylenetetrahydrofuran was synthesized from 2-(bromomethyl)-tetrahydrofuran by treatment with potassium hydroxide, in moderate yields (47%) according to Armitage et al. [30]. The formed 2-methylenetetrahydrofuran was used in a Diels-Alder reaction with methyl vinyl ketone in analogy with Ireland and Häbich [31], yielding the product 7-methyl-1,6-dioxaspiro[4.5]dec-7-ene (DHC) in 63% yield (Fig. 2).

### Trial 3: Test of DHC

The inhibitory effect of DHC on pheromone trap catch was evaluated in Trial 3 and compared to the inhibitory effect of pure tC. A total of 2 812 beetles were caught in 15 replicates. Also in this trial catches among the traps, including a pheromone only control and three inhibitory treatments, differed significantly by ANOVA (F = 32.4; df = 3; p<0.001) followed by Tukey HSD on arcsin√(relative catch). The trap with pheromone alone caught significantly more beetles than the other treatments (p<0.001) (Fig. 5). The treatment including tC in combination with 1-hexanol and (−)-verbenone had significantly lower catch than the treatment including DHC (instead of tC) (p<0.05). The treatment with tC also had a significantly lower trap catch (p<0.001) than the treatment with only (−)-verbenone and 1-hexanol as anti-attractants, whereas this was not the case for the treatment with DHC (instead of tC) (p = 0.69).

### Single-sensillum Recordings

**Figure 6:**
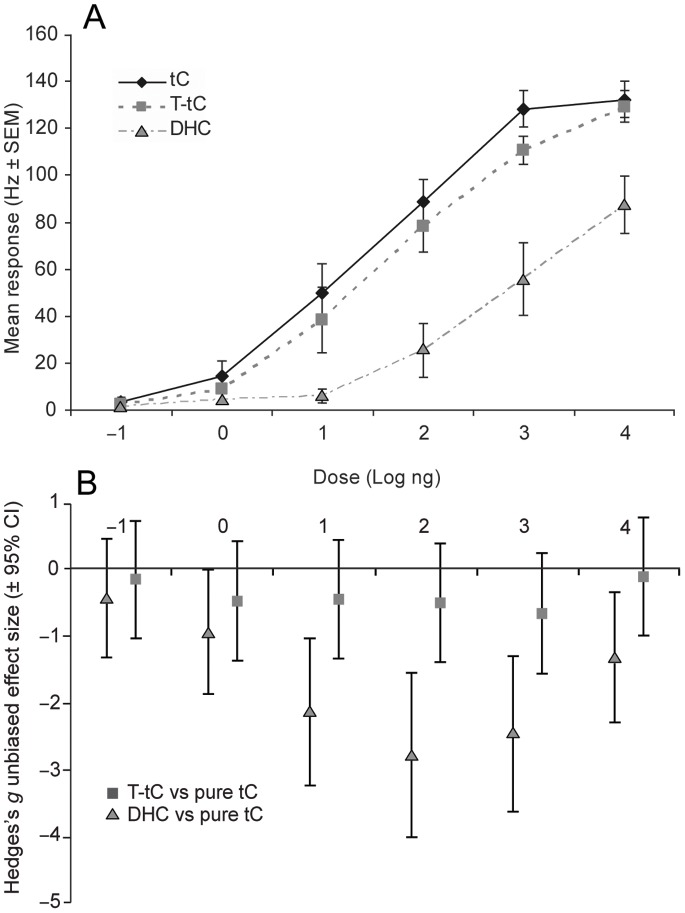
Single sensillum recordings. **A)** Dose-response curves recorded from the *trans*-conophthorin (tC) ORN class [23], demonstrating the highest sensitivity to the pure tC, and somewhat lower sensitivity to the technical grade tC (T-tC). The lowest sensitivity was recorded to dehydro-conophthorin (DHC) (*n = *10, i.e. 5 sensilla from each sex). **B)** The physiological effect of T-tC and DHC (expressed as Hedges’ *g* effect size) in relation to pure tC that served as positive control in the analysis. Negative effect sizes indicate weaker responses to T-tC and DHC than to pure tC, as also seen in **A**. Responses with CI:s below zero were regarded as significantly weaker (p<0.05) than the response to pure tC at the specified dose.

To provide a mechanistic explanation for the similar reduction in trap catch by the presence of T-tC and tC, but lower reduction by DHC, SSR from the *trans*-conophthorin sensitive ORN class were performed. The tC neuron responded the strongest and was most sensitive to pure tC (Fig. 6A). The response threshold for this stimulus was below the 1 ng dose. Somewhat weaker responses, but not significantly different (see below), were elicited by T-tC, which probably only reflected the lower purity of the compound. DHC also elicited responses, but the sensitivity to this compound was almost 100 times lower. According to the effect size analysis, the responses to T-tC were not significantly different from those elicited by pure tC at any dose (i.e. CI:s at all doses cross zero; Fig. 6B). In contrast, DHC had significantly lower physiological effects than pure tC (CI:s below zero) at all doses, except for the lowest (100 pg) dose where none of the compounds elicited responses. The largest difference in effect between pure tC and DHC was found at the 100 ng dose (*g* = −2.77).

## Discussion

Our results from three years of field trials demonstrate that the previously reported most potent anti-attractant mixture for *I. typographus* for forest protection applications, containing verbenone and four non-host volatile compounds [4] can be simplified and made more cost effective by removing the two unsaturated GLV compounds (3*Z*)-hexen-1-ol and (2*E*)-hexen-1-ol, and releasing 1-hexanol alone at the rate of the whole blend (6 mg/day) (Trial 1). Furthermore, technical grade tC (T-tC) could replace the expensive high purity product and doses lowered from 5 mg/day to 0.5 mg/day, which would reduce the costs even further (Trial 2). In Trial 1 with relatively lower synthetic pheromone release rate, the release rate (of pure tC) could actually be reduced 100-fold (to 0.05 mg/day) while still maintaining a significant effect, but this lowest dose was insufficient to achieve a significant effect in Trial 2 with a different pheromone attractant and a higher beetle activity, in spite of more replicates. Thus, for forest protection purposes, it seems unadvisable to use tC or T-tC at release rates below 0.5 mg/day. Fortunately, impurities in the T-tC as well as the presumably slightly lower release of the tC itself from the technical grade preparation did not seem to influence the anti-attractant properties. In contrast to T-tC, the dehydro-conophthorin (DHC) analog clearly had a lower activity, and is thus hardly suitable for management of *I. typographus* (Trial 3). DHC differs from tC only by the presence of one double bond, suggesting a high specificity of the olfactory sense of *I. typographus.*

To provide a mechanistic explanation for the lower activity of DHC, we performed single sensillum recordings from the previously characterized tC ORN class [23]. This neuron was clearly less sensitive to DHC than to tC. Since the tC-responsive ORN is the main olfactory input channel for tC [23], we believe that its lower sensitivity is likely to explain the lower effectiveness of DHC in reducing pheromone trap catch. Not surprisingly, the T-tC elicited physiological responses that were similar to those elicited by the pure tC. Previously, two other compounds (chalcogran and *exo*-brevicomin) that are also structurally similar to tC were tested on this neuron class [23]. Although these two compounds elicited stronger responses than DHC, they were still less active than tC, again demonstrating a high selectivity of this neuron to this specific non-host volatile compound [20]. Correspondingly, one of the pioneering SSR studies on *I. typographus* demonstrated that the neurons that are selective for (+)- and (−)-ipsdienol, respectively, clearly displayed weaker responses to several structurally similar analogs, indicating a high selectivity also for these two neuron classes [32]. Specific input channels for anti-attractant or repellent compounds have been found also in other species. For instance, a recent study demonstrated an extremely specific “labeled line” channel for the repellent compound geosmin in *Drosophila melanogaster*, signaling harmful microbes and spoiled food [33].

In contrast to the specific tC ORN class, *I. typographus* carries another neuron class that previously was found not to discriminate between the three anti-attractant GLV alcohols, 1-hexanol, (3*Z*)-hexen-1-ol and (2*E*)-hexen-1-ol [23]. Thus, again there is a likely mechanism in the peripheral olfactory sense explaining the behavior (reduction in trap catch), i.e. compounds could be “behaviorally redundant” [4] if the odorant receptors [34] or ORNs cannot tell them apart (as also discussed in Andersson et al., [23]). In contrast to tC, GLVs are found ubiquitously in green tissue of angiosperm plants and for a conifer-feeding insect it is plausible that there is no need for specific ORNs for different compounds that convey the same message (redundant information), i.e. green leaves on non-host plants.

In North America, pine attacking bark beetles of the *Dendroctonus* genus can be managed using verbenone as a single anti-attractant [17], [35]–[37]. Verbenone has been shown to be released by both sexes in *Dendroctonus* spp. and has been proposed as an “antiaggregative-rivalry” pheromone [38], but also the importance of microbial interconversion and autoxidation in this process has been pointed out [39]. On the other hand, in *I. typographus* there is no evidence that verbenone is released by the beetle itself [40], but the compound has been shown to be produced in gallery walls by yeasts [11], [12]. Thus, whereas verbenone seems to act as a potent anti-attractant for *Dendroctonus* spp., it only moderately modulates pheromone attraction of *I. typographus*. Fortunately, its anti-attractant effect can be greatly synergized by combining the signal with general (GLVs) and specific (*trans*-conophthorin) non-host volatiles. However, also in *Dendroctonus brevicomis* the addition of various non-host volatiles augmented the inhibition to pheromone by verbenone [41]. In *I. typographus*, we confirm the results from previous studies [3], [5] that the males, the host-choosing sex, were inhibited more by verbenone, and even more by additional NHV, than the females.

Based on the results from the present study, we thus propose a ternary blend consisting of (–)-verbenone, 1-hexanol and technical grade *trans*-conophthorin as a cost-efficient anti-attractant in operational forest protection strategies against *I. typographus*. The application of verbenone with its relatively low volatility in forest management offers the possibility of area-wide dispensing techniques [35], whereas the necessity to use NHV with higher volatility in European forests still requires optimization of dispensing techniques using the proposed anti-attractant blend.
